# Economic evaluation of legislation-based public health interventions – the case of tobacco control

**DOI:** 10.1093/eurpub/ckac129.235

**Published:** 2022-10-25

**Authors:** J Perelman, T Leão, M Serrano-Alarcon

**Affiliations:** 1 NOVA National School of Public Health, NOVA University of Lisbon, Lisbon, Portugal; 2 Comprehensive Health Research Center, NOVA University of Lisbon, Lisbon, Portugal; 3 EPIUnit - Instituto de Saúde Pública, Universidade do Porto, Porto, Portugal; 4 Faculdade de Medicina, Universidade do Porto, Porto, Portugal; 5 DONDENA, Bocconi University, Milan, Italy

## Abstract

Economic evaluation of public health interventions have mostly been performed for interventions whose cost and effectiveness can be well identified, such as protective interventions (vaccination or screening), clinical interventions (prevention drugs) or counselling and education programs. Yet, following the Thomas Frieden's health impact pyramid, these public health interventions are less effective than those “changing the context to make individuals’ default decision healthy”. The economic evaluation of population-based interventions that modify the context is rather challenging. First, such interventions include various components, e.g., taxes and subsidies, media campaigns or bans. Second, since they correspond to political decisions applied at a wide scale, their effectiveness can hardly be measured using experimental designs. Third, the cost of such interventions is difficult to assess, since they are not represented by a specific product or service, but rather by political decisions and their implementation, whose costing is not straightforward. Using the case of tobacco control policies, we show how alternative methods can be used to assess their economic value. We show that quasi-experimental methods with country comparisons allow identify the effectiveness of specific policies, and that qualitative approaches are needed to quantify their costs. Results indicate that tobacco-control policies have low costs and are highly effective, so that their cost-effectiveness is quite favorable in light of commonly referred thresholds, and in comparison with widely financed clinical interventions. Population-based interventions that focus contextual factors must also be carefully evaluated from an economic viewpoint. Although economic evaluations are challenging, alternative approaches help obtain results that are valuable for decision making.

